# High expression of microRNA-155 is associated with the aggressive malignant behavior of gallbladder carcinoma

**DOI:** 10.3892/or.2013.2443

**Published:** 2013-05-09

**Authors:** HIROSHI KONO, MASAFUMI NAKAMURA, TAKAO OHTSUKA, YOSUKE NAGAYOSHI, YASUHISA MORI, SHUNICHI TAKAHATA, SHINICHI AISHIMA, MASAO TANAKA

**Affiliations:** 1 Department of Surgery and Oncology, Graduate School of Medical Sciences, Kyushu University, Fukuoka 812-8582, Japan; 2 Department of Anatomic Pathology, Graduate School of Medical Sciences, Kyushu University, Fukuoka 812-8582, Japan; 3 Department of Digestive Surgery, Kawasaki Medical School, Okayama 701-0192, Japan

**Keywords:** gallbladder cancer, pancreaticobiliary maljunction, microRNA-155

## Abstract

The prognosis of gallbladder cancer (GBC) remains poor despite recent advances in diagnostics and therapeutic strategies. Although the role of microRNAs (miRs) in GBC have not been well documented, miR-155 is known to be associated with inflammation-associated carcinogenesis in various types of cancers. The aim of this study was to investigate the clinical significance of miR-155 expression and the biological functions of miR-155 in GBC. The expression levels of miR-155 in surgically resected GBCs and gallbladders with pancreaticobiliary maljunction (PBM) were assessed by quantitative reverse transcription-polymerase chain reaction. The relationship between the expression levels of miR-155 and clinicopathological features of GBCs was analyzed. Human GBC cell lines were transfected with miR-155 inhibitors or mimics, and the effects on proliferation and invasion were assessed. miR-155 was significantly overexpressed in GBCs when compared with that in gallbladders with PBM (P=0.007) and normal gallbladders (P=0.04). The high expression level of miR-155 in GBCs was significantly associated with the presence of lymph node metastasis (P=0.01) and a poor prognosis (P=0.02). *In vitro* assays showed that aberrant expression of miR-155 significantly enhanced GBC cell proliferation and invasion. In conclusion, high miR-155 expression correlates with the aggressive behavior of GBCs, and miR-155 may become a prognostic marker and therapeutic target for GBC.

## Introduction

The prognosis of gallbladder cancer (GBC) remains poor despite recent advances in diagnostic modalities and therapeutic tools. The incidence of GBC is >4,000 cases per year, and the 5-year survival rate of patients with GBC is estimated to be ~12% (1). The poor prognosis of GBC appears to be associated with the absence of specific symptoms, which causes difficulty for the early diagnosis of GBC. Indeed, most early-stage GBCs are found incidentally after cholecystectomy for cholecystolithiasis, of which the reported incidence is ~0.3–1% (2,3). Therefore, novel diagnostic methods and therapeutic agents are needed to improve the prognosis of GBC.

Pancreaticobiliary maljunction (PBM) is a major risk factor for GBC and is one of the clues used to clarify the mechanisms of carcinogenesis and malignant behavior of GBCs. Reflux of pancreatic juice into the bile duct activates the conversion of lecithin to lysolecithin by phospholipase A2, and concentrated lysolecithin in the gallbladder or the dilated bile duct induces chronic inflammation and damage to biliary epithelium (4). K-ras and p53 mutations as well as MUC1 overexpression have been frequently detected in GBC with PBM (5–8). It has been speculated that K-ras mutation and chronic inflammation induce mucosal hyperplasia in the biliary system, and the subsequent p53 mutation causes lesions to develop atypical hyperplasia and then transform to carcinoma. The ‘hyperplasia-to-atypical hyperplasia-to-carcinoma sequence’ in GBC with PBM differs from the ‘adenoma-to-carcinoma sequence’ or ‘*de novo*’ oncogenesis (7–9). Thus, elucidation of this carcinogenic process may help us to establish novel diagnostic and therapeutic tools for GBC.

microRNAs (miRs) are non-coding RNAs consisting of 18–25 nucleotides, which have been reported to play important roles in the regulation of carcinogenesis and cancer progression as well as homeostasis. Approximately 1500 miRs have been identified to date, and aberrant expression of miRs has been detected in various types of malignancies including breast, colorectal and lung cancers (10,11). Although the effects of aberrant regulation of miRs on malignant diseases have not been fully explored, miRs are expected to become diagnostic markers or therapeutic targets for various malignancies including GBCs.

Previous reports have shown that miR-155 is involved in the carcinogenesis of B-cell lymphoma, breast, lung and pancreatic cancers (12–16). Jiang *et al*(15) speculated that the mechanisms of inflammatory stimulation through the Janus-activated kinase pathway may cause upregulation of miR-155 and subsequently lead to mutation of tumor-suppressor gene SOCS1 in breast cancer. Tili *et al*(17) reported that miR-155 overexpression and an inflammatory environment in breast cancer cell line MDA-MB-21 downregulated WEE-1 kinase, which blocks cell cycle progression. The authors concluded that miR-155 is involved in inflammation-related carcinogenesis and cancer progression. To date, despite the recent findings concerning miR-155 and its important roles in inflammation and carcinogenesis, there have been no reports regarding an association between miR-155, GBCs and PBMs.

The aims of this study were to explore the expression level of miR-155 and evaluate the clinical significance of miR-155 expression in GBC and non-cancerous gallbladder samples with the presence or absence of PBM, and to evaluate the effects of aberrant miR-155 expression on GBC cell lines.

## Materials and methods

### Tissue sampling

**Figure 1:**
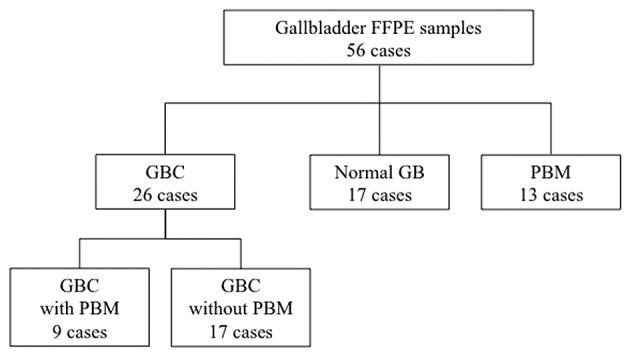
Flow diagram of the patients in this study. GBC, gallbladder cancer; GB, gallbladder; PBM, pancreaticobiliary maljunction; FFPE, formalin-fixed paraffin-embedded.

Samples were obtained from 56 patients who underwent surgical resection of gallbladders at our institution between January 1994 and June 2011. There were 17 GBCs without PBM, 9 GBCs with PBM, and 13 non-cancerous gallbladders with PBM (Fig. 1). Normal gallbladders obtained from 17 patients who underwent pancreatoduodenectomy for intraductal papillary mucinous neoplasm were used as controls. Formalin-fixed, paraffin-embedded (FFPE) blocks of gallbladders were sectioned at 10-μm thicknesses, and the epithelia of GBCs, non-cancerous gallbladders with PBM, and normal gallbladders were obtained. Cancerous lesions of GBCs were obtained by histological macrodissection with manual scalpel resection while referring to hematoxylin and eosin staining. The study was approved by the Ethics Committee of Kyushu University and was conducted according to the Ethical Guidelines for Human Genome/Gene Research enacted by the Japanese government and the Helsinki Declaration.

### RNA extraction from FFPE tissues

Total RNA was purified from FFPE tissues of the gallbladder epithelium as described above using an RNeasy FFPE kit (Qiagen, Hilden, Germany) in accordance with the manufacturer's protocol. The RNA quantity was measured with an ND-1000 spectrophotometer (Thermo Fisher Scientific, Wilmington, DE, USA). Total RNA was checked by a 2100 Bioanalyzer (Agilent Technologies, Santa Clara, CA, USA) to determine the RNA quality integrity number and 28s/18s rRNA ratio.

### miR-155 expression analysis by real-time reverse transcription-polymerase chain reaction

Purified RNAs were subjected to miR assay. A stem-loop quantitative real-time reverse transcription-polymerase chain reaction (qRT-PCR) was performed with a TaqMan MicroRNA Reverse Transcription kit and TaqMan Universal PCR Master Mix II (Applied Biosystems, Foster City, CA, USA) and Chromo4 real-time PCR detection system (Bio-Rad Laboratories, Hercules, CA, USA) for each sample according to the manufacturer's instructions. cDNAs were placed in triplicate onto a plate in a total volume of 5 μl/well. Briefly, for reverse transcription, the reaction mixtures were incubated at 16°C for 30 min, 42°C for 30 min, 85°C for 5 min, and then maintained at 4°C. PCR amplification was performed at 95°C for 10 min, followed by 40 cycles at 94°C for 15 sec and 60°C for 60 sec. The expression levels of specific miRs were normalized to those of RNA U6 (RNU6B) as the endogenous control.

### Cell lines

Human GBC cell lines G-415 (isolated from GBC of a 68-year-old Japanese male), OCUG-1 (isolated from GBC of a 43-year-old Japanese male), and NOZ (isolated from ascites of a 48-year-old Japanese female with GBC) were used in the present study. The G-415 cell line was purchased from the Riken BioRecourse Center through the National Bio-Resource Project of the Ministry of Education, Science, Sports Culture and Technology (Tsukuba, Japan), and maintained in Roswell Park Memorial Institute (RPMI)-1640 medium supplemented with 10% fetal bovine serum (FBS). OCUG-1 and NOZ cell lines were purchased from the Japan Health Science Research Resources Bank (Osaka, Japan), and maintained in Dulbecco's modified Eagle's medium (DMEM) with 10% FBS and Williams' E medium with 10% FBS, respectively. Each cell line was cultured at 37°C in a humidified incubator with 5% CO_2_.

### Analysis of miR-155 expression levels in GBC cell lines

To analyze the expression levels of miR-155 in the GBC cell lines by qRT-PCR, total RNA was extracted from each GBC cell line using a mirVana miRNA Isolation kit (Applied Biosystems) according to the manufacturer's instructions. qRT-PCR was performed using a Chromo4 real-time PCR detection system with TaqMan microRNA assays (Applied Biosystems) as described above.

### Transfections

The cell lines were transfected by electroporation using an Amaxa Biosystems Nucleofector (Lonza, Basel, Switzerland) according to the manufacturer's protocol. Briefly, 100 pmol of the inhibitors or mimics of miR-155 or a negative control miR was added to 1×10^6^ cells suspended in 100 μl Nucleofector solution, followed by electroporation. The sequence of the mimic for miR-155 was 5′-UUAAUGCUAAUC GUGAUAGGGGU-3′. The sequence of the inhibitor for miR-155 was 5′-UCCCCTUTCUCGUTTUGCUTTUU-3′. Negative control miR was used as described by Gregory *et al*(18). Synthesized RNAs were all purchased from Qiagen. After electroporation, transfected cells were seeded in 90-mm dishes and cultured for 24 h at 37°C, and then viable cells were collected and applied to proliferation and invasion assays. Effects of the miR-155 inhibitors and mimics, and the negative control on GBC cell lines were evaluated with the TaqMan microRNA assay.

### Proliferation assay

Cell proliferation was assessed by a modified propidium iodide (PI) assay as previously described (19). Briefly, after 24 h of transfection, 2×10^4^ cells/well were seeded in 24-well plates containing medium without phenol-red and then incubated at 37°C for 48 and 96 h. At the indicated time, 30 μM PI and 600 μM digitonin were added to each well. After incubation for 30 min at 37°C, the fluorescence intensity was measured by an Infinite 200 multi-well plate reader (Tecan, Männedorf, Switzerland) with 530-nm excitation and 645-nm emission filters, and the total number of cells was calculated. Each experiment was performed three times in triplicate.

### Matrigel invasion assay

Cancer cell invasion was assessed by measuring the movement of cells into an artificial basal membrane in Matrigel-coated Transwell chambers (Becton-Dickinson, Franklin Lakes, NJ, USA). Each insert had an 8-μm pore-sized membrane coated with 20 μg Matrigel. Briefly, 750 μl culture medium was placed into each well of a 24-well plate, and then 1×10^5^ cells suspended in 250 μl culture medium were seeded into each insert. After 16 h of incubation at 37°C with 5% CO_2_, the non-invading cells on the surface of the membrane were removed by scrubbing with a cotton swab. The invading cells on the lower surface of the membrane were fixed with 70% ethanol and stained with hematoxylin and eosin. The invading cells in five randomly selected fields were counted under a light microscope at ×100 magnification. Each experiment was performed three times in triplicate. Cell growth of GBC cells used in the invasion assays was not observed after 16 h.

### Statistical analysis

The cut-off value of miR-155 expression was set by the median value of the expression levels, and the lesions were divided into two groups according to high and low expression levels of miR-155. The relationship between the expression level of miR-155 and clinicopathological characteristics, including prognosis, was assessed. The effect of possible prognostic factors on survival was also analyzed. Comparison between two groups was analyzed with the Chi-square test, Mann-Whitney U test, or Cox regression test. Cumulative survival rates were calculated with the Kaplan-Meier method and compared with the log-rank test using JMP version 9.0.2 (SAS Institute, Inc., Cary, NC, USA). Differences were considered significant when the probability value was <0.05.

## Results

### Overexpression of miR-155 in GBCs

**Figure 2:**
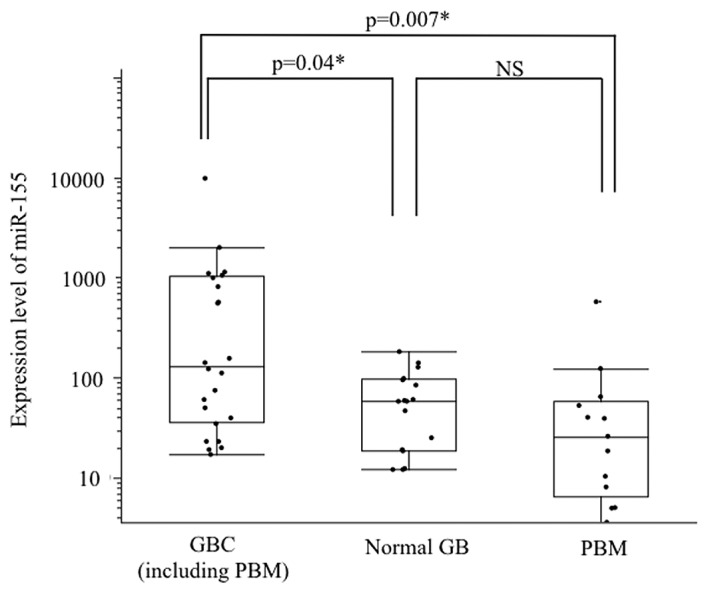
Analysis of the miR-155 expression level in GBCs, gallbladders with PBM, and normal gallbladders by qRT-PCR. miR-155 levels were normalized to RNU6B expression as an internal control. The expression level of miR-155 in GBCs was significantly higher than that in gallbladders with PBM (P=0.007) or normal gallbladders (P=0.04). There was no significant difference in the miR-155 expression level between gallbladders with PBM and normal gallbladders. miR, microRNA; GBC, gallbladder cancer; GB, gallbladder; PBM, pancreaticobiliary maljunction.

The expression level of miR-155 was examined in 26 GBCs (including 9 with PBM), 13 non-cancerous gallbladders with PBM, and 17 normal gallbladders by qRT-PCR (Fig. 1). The expression level of miR-155 was significantly higher in the GBCs when compared with that in the normal gallbladders, whereas miR-155 was not upregulated in gallbladders with PBM (Fig. 2). High expression of miR-155 (>1.50-fold) was more frequently observed in GBCs (14/26, 53%) than that in normal gallbladders (4/17, 23%) (P=0.01). There was no significant difference in the miR-155 expression level between GBCs with PBM (n=9) and GBCs without PBM (n=17) (P=0.12). There was also no significant difference in the miR-155 expression level between GBCs with PBM (n=9) and non-cancerous gallbladders with PBM (n=13) (P=0.08).

### Relationship between the expression level of miR-155 and clinicopathological features in patients with GBC

High miR-155 expression was significantly associated with the presence of lymph node metastasis (P=0.03) and vessel invasion (P=0.007) (Table I). There was no significant difference in age, gender, presence of PBM, T categories, UICC stages, lymphatic invasion, or perineural invasion between high and low levels of miR-155 expression.

### Relationship between the expression level of miR-155 and prognosis in patients with GBCs

**Figure 3:**
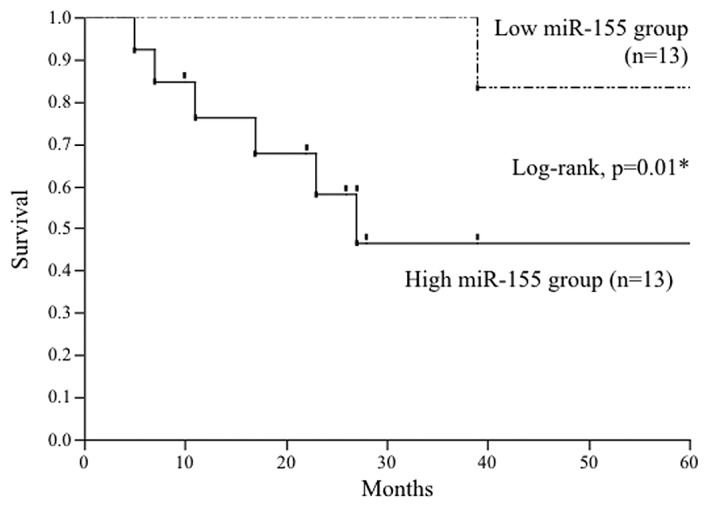
Effect of the expression level of miR-155 on the disease-specific survival rate of patients with GBC. The cut-off level was set at the median value of the miR-155 expression levels in 26 patients with GBC. The high miR-155 group showed a poorer prognosis than that of the low miR-155 group (P=0.01). miR, microRNA; GBC, gallbladder cancer.

The relationship between the expression level of miR-155 and the disease-specific survival (DSS) rate after surgery was examined. DSS was significantly lower for GBC patients with a high expression level of miR-155 (5-year survival rate, 35%; median, 25 months) than those with a low miR-155 expression level (5-year survival rate, 85%; median, 45 months) (P=0.01) (Fig. 3). The presence of lymph node metastasis (P<0.0001), advanced UICC stage (IIB-IV) (P=0.03) and positive lymphatic invasion (P=0.005) were also significantly associated with poor prognoses of GBC patients, whereas the presence of PBM, T category (Tis-2 vs. T3–4), vessel invasion and perineural invasion did not affect the prognosis of GBC patients (Table II). Multivariate analysis revealed that lymphatic invasion was the only significant independent factor to predict prognosis (hazard ratio, 9.6; confidence interval, 1.3–189; P=0.03) (Table III). However, when focusing on GBCs localized around the gallbladder without lymph node metastasis (stage 0-IIA of UICC, Tis-T3N0), high miR-155 expression (P=0.02), lymphatic invasion (P=0.03) and vessel invasion (P=0.02) were significant predictive factors for the prognoses of GBC patients as indicated by univariate analysis. Multivariate analysis showed that a high expression level of miR-155 was the only independent predictive indicator for poor prognosis of patients with localized GBCs (hazard ratio, 9.9; confidence interval, 1.10–294; P=0.03) (Table IV).

### Expression levels of miR-155 in GBC cell lines and the effect of transfection on the expression level of miR-155

**Figure 4:**
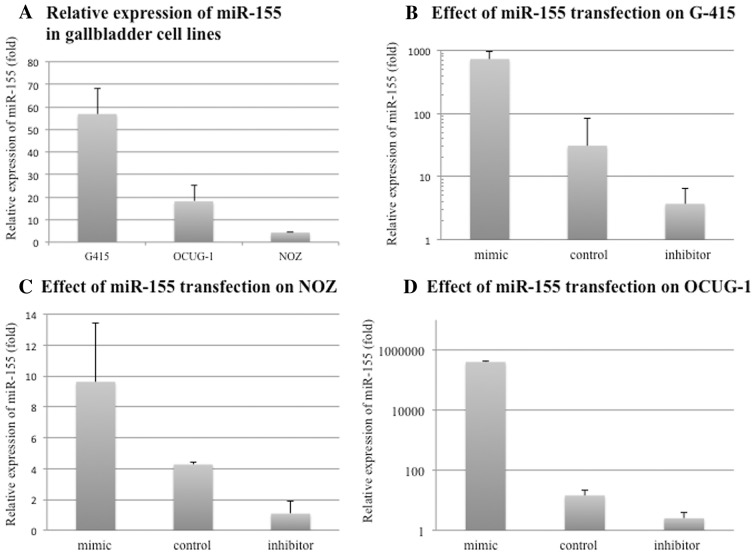
Relative expression of miR-155 in GBC cell lines and the effect of transfection on the miR-155 expression level. (A) The expression levels of miR-155 in the GBC lines were investigated by qRT-PCR. miR-155 expression levels were normalized to RNU6B expression. Human GBC cell lines, G-415 (isolated from a GBC of a 68-year-old Japanese male), OCUG-1 (isolated from the GBC of a 43-year-old Japanese male), and NOZ (isolated from the ascites of a 48-year-old Japanese female with GBC), were used in the present study. miR-155 expression levels in G-415, NOZ and OCUG-1 cells were 56.8-, 18.1- and 4.2-fold, respectively. (B-D) Effects of transfection of miR-155 inhibitors or mimics on each GBC cell line were assessed by qRT-PCR. Each expression level of miR-155 was normalized to RNU6B expression. Expression levels of miR-155 in G-415, NOZ and OCUG-1 cells transfected with miR-155 inhibitors were downregulated by 0.12-, 0.35- and 0.17-fold, respectively, when compared with levels in the negative controls. The expression levels of miR-155 in G-415, NOZ and OCUG-1 cells transfected with miR-155 mimics were upregulated by 23.6-, 124- and 27,605-fold, respectively, when compared with levels in the negative controls. miR; microRNA.

The relative expression levels of miR-155 to RUN6B expression in G-415, NOZ and OCUG-1 cells were 56.8-, 18.1- and 4.2-fold, respectively (Fig. 4A). Expression levels of miR-155 in G-415, NOZ, and OCUG-1 cells transfected with miR-155 inhibitors showed downregulation by 0.12-, 0.35- and 0.17-fold, respectively, when compared with levels in the negative controls. Expression levels of miR-155 in G-415, NOZ and OCUG-1 cells transfected with miR-155 mimics were upregulated by 23.6-, 124- and 27,605-fold, respectively, when compared with levels in the negative controls (Fig. 4B-D).

### Aberrant miR-155 expression affects the proliferation of GBC cell lines

**Figure 5:**
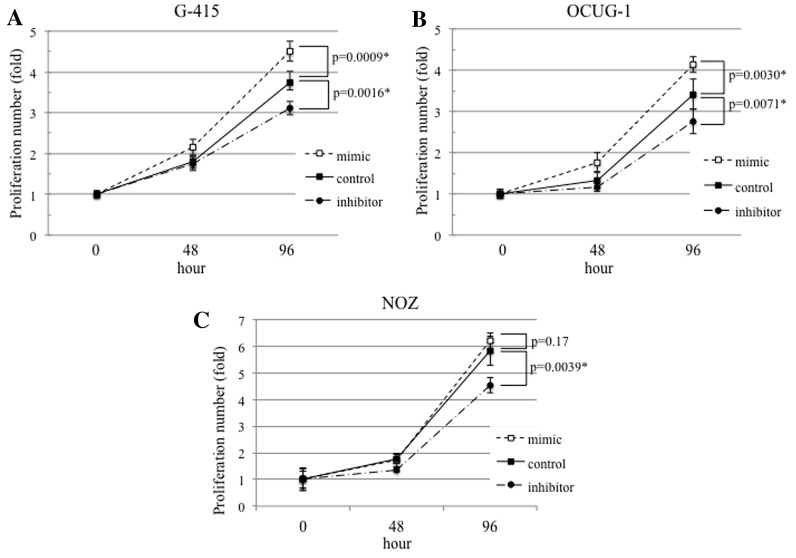
Effects of transfection with miR-155 inhibitors or mimics on the proliferation of GBC cell lines. The effects of transfection with miR-155 inhibitors or mimics on the proliferation of GBC cell lines were assessed after 48 and 72 h of incubation. (A) G-415 cells showed significant increases (P=0.0009) or decreases (P=0.0016) in cell proliferation after transfection with miR-155 mimics or inhibitors, respectively. (B) OCUG-1 cells also showed significant increases (P=0.0030) or decreases (P=0.0071) in cell proliferation after transfection with miR-155 inhibitors or mimics, respectively. (C) NOZ cells transfected with miR-155 inhibitors showed decreased cell proliferation (P=0.0039), while those transfected with miR-155 mimics showed no significant differences in cell proliferation (P=0.17). Error bars represent the standard deviation. miR; microRNA.

G-415 (P=0.0016) and OCUG-1 (P=0.0071) cells transfected with miR-155 inhibitors showed significant decreases in cell proliferation, compared with that in the negative controls. On the other hand, G-415 (P=0.0009) and OCUG-1 (P=0.0030) cells transfected with miR-155 mimics showed significant increases in cell proliferation, compared with that in the negative controls (Fig. 5A and B). NOZ cells transfected with miR-155 inhibitors showed decreased cell proliferation (P=0.0039), whereas those transfected with miR-155 mimics showed no significant differences in cell proliferation (P=0.17) (Fig. 5C).

### Aberrant miR-155 expression affects the invasion of GBC cell lines

**Figure 6:**
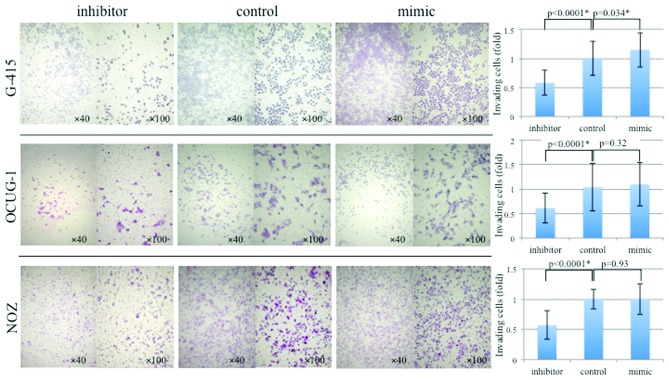
Effects of transfection with miR-155 inhibitors or mimics on the invasive ability of GBC cell lines. G-415, OCUG-1 and NOZ cells were transfected with miR-155 inhibitors or mimics, and their invasiveness was assessed after 16 h of incubation. The number of invasive cells among the total number of G-415 cells transfected with miR-155 mimics was significant higher (P=0.034) compared with that of the normal control, whereas no difference was observed in the OCUG-1 (P=0.32) and NOZ (P=0.93) cells. The numbers of invasive cells among the total numbers of G-415, NOZ and OCUG-1 cells transfected with miR-155 inhibitors were significantly lower (all P<0.0001) compared with those of the normal controls. Error bars represent the standard deviation. Magnifications, ×40 and ×100 for left and right panels, respectively.

In the invasion assay, the number of invasive cells among the total number of G-415 cells transfected with miR-155 mimics was significantly higher compared with that of the normal control (P=0.034), whereas no difference in invasion was observed in OCUG-1 (P=0.32) and NOZ (P=0.93) cells transfected with mimics. The numbers of invasive cells among G-415, NOZ and OCUG-1 cells transfected with miR-155 inhibitors were significantly lower (all P<0.0001) compared with those of the normal controls (Fig. 6).

## Discussion

In the present study, we found that: i) miR-155 is upregulated in GBCs irrespective of the presence or absence of PBM, ii)high miR-155 expression is significantly associated with the presence of lymph node metastasis and vessel invasion, and indicates a poorer prognosis for GBC patients, when compared with GBC patients with low miR-155 expression, and iii) upregulation of miR-155 affects the proliferation and invasion of GBC cells *in vitro*. To our knowledge, this is the first report demonstrating aberrant miR-155 expression and its effects on the function of GBC cells.

One of the clinical problems during management of GBCs is that many GBCs are found at an advanced stage with metastasis at the time of diagnosis, and most early-stage GBCs within the mucosal layer are diagnosed incidentally after cholecystectomy for cholecystolithiasis. Because of the difficulty of the early detection of GBCs, identification of sensitive markers to diagnose early-stage GBCs is urgently needed. Our present study was planned based on the hypothesis that assessment of abnormal regulation of miRs may help us to understand the complicated mechanism of carcinogenesis and address the clinical problems of GBC.

Recent reports have analyzed the relationship between the expression level of miR-155 and the prognosis of several types of cancers. Shibuya *et al*(20) showed that high expression of miR-155 in colorectal cancer is associated with a high incidence of lymph node metastasis and poor prognosis. The relationship between miR-155 upregulation and advanced stages with poor prognoses has also been reported in lung cancers (14). Our present study also demonstrated that the expression level of miR-155 can predict the prognosis of stage 0-IIA GBCs.

Stable miRs have been recently found in human blood, despite the presence of RNase, and abnormal regulation of miRs has been detected in the serum of patients with breast, esophageal and prostate cancers (21–24). Henegham *et al*(22) identified upregulated miR-195 and let-7a in the serum of breast cancer patients while decreased levels of these miRs after surgery were noted. These circulating miRs were also correlated with clinicopathological factors such as nodal and estrogen receptor statuses. miR-155 has been detected in the serum of patients with diffuse large B-cell lymphoma and is expected to become a diagnostic marker for this cancer (25,26). Recently, Shigehara *et al*(27) demonstrated that miR-9 and miR-145 are present in bile samples, and aberrant expression of these miRs in bile may be a diagnostic marker for hepatobiliary tract cancers. Further examination targeting miR-155 in serum and/or bile might help us to develop diagnostic markers for early detection of GBCs. In addition, detecting upregulation of miR-155 in the serum and/or bile of patients with gallbladder disease will be useful to determine surgical procedures when it is difficult to distinguish GBC from benign gallbladder diseases such as xanthogranulomatous cholecystitis.

miRs are also expected to become molecular targets of cancer therapy (28). Garzon *et al*(29) showed that upregulated miR-155 causes the development of acute lymphocytic lymphoma and that silencing miR-155 using antisense oligonucleotides inhibits this process. Xie *et al*(30) found that ectopic expression of miR-155 in hepatocellular carcinoma cells enhances *in vitro* cell proliferation by targeting the transcriptional regulator, sex-determining region Y box 6. Our present study also demonstrated that aberrant expression levels of miR-155 by transfection of miR-155 inhibitors or mimics, were correlated with proliferation and the invasiveness of GBC cell lines. In particular, G-415 cells with an unmodified high expression level of miR-155 showed marked inhibition or promotion of proliferation and invasion following transfection of miR-155 inhibitors or mimics. These results are in good agreement with the hypothesis that miR-155 is a regulator of proliferation and invasion of GBC cells. The present study suggests the possibility that modulation of the miR-155 level may be applied to the treatment of GBCs, particularly for inhibition of cancer progression such as lymph node invasion.

In contrast to our expectation, PBM did not affect regulation of the miR-155 level in gallbladder epithelium. This finding suggests that miR-155 is not involved in the early stage of multistep carcinogenesis induced by inflammation, the ‘hyperplasia-to-atypical hyperplasia-to-carcinoma sequence’. Furthermore, it appears that the penetration frequency of miR-155 is too low to be a candidate for a general factor involved in carcinogenesis. miR-155 may be overexpressed in the process in which established cancers acquire invasive character.

Evaluation of GBCs as well as gallbladders with PBM by microarray may reveal the association of miRs with multistep carcinogenesis under chronic inflammation. miRs are characterized by their binding to the incomplete complementary sites of their targets and allowing mismatched G-U base pairing. However, a single gene target is regulated by several miRs (12). Therefore, it is be necessary to analyze potential aberrant expression of several miRs at the same time to elucidate their effects on carcinogenesis and cancer progression.

In conclusion, high miR-155 expression correlates with the aggressive behavior of GBCs, and miR-155 may become a prognostic marker and therapeutic target for GBC.

## Figures and Tables

**Table I:** Correlation between clinicopathological features and the miR-155 expression level in GBCs.

Clinicopathological features	Total	High expression	Low expression	P-value
Total no. of GBC cases	26	13	13	
Mean age, years ± SD (range)	65±11 (42–85)	66±11 (45–85)	64±11 (42–83)	0.66
Gender, male/female (%)	11 (42)/15 (58)	8/5	6/7	0.67
Presence of PBM (%)	9 (35)	3	6	0.97
T category (UICC) (%)				
Tis/T1/T2	3 (12)/4 (15)/16 (61)	0/1/11	3/3/5	0.56
T3/T4	1 (4)/2 (8)	0/2	1/0	
N category, N1/N0 (%)	5 (19)/21(81)	5/8	0/13	0.03^a^
UICC stage (%)				
0/IA/IB/IIA	3 (12)/4 (15)/13 (50)/0 (0)	0/1/8/0	3/3/5/0	0.11
IIB/III/IV	4 (15)/2 (8)/0 (0)	3/2/0	1/0/0	
Lymphatic invasion (%)				
Positive	13 (50)	9	4	0.11
Negative	13 (50)	4	9	
Vessel invasion (%)				
Positive	13 (50)	9	4	0.007^a^
Negative	13 (50)	3	10	
Perineural invasion (%)				
Positive	13 (50)	5	8	0.11
Negative	13 (50)	1	12	
Follow-up period				
Median ± SD (months)	35±33	25±19	46±40	0.29
5-year DSS rate	0.62	0.46	0.83	0.01^a^

miR, microRNA; GBC, gallbladder cancer; PBM, pancreaticobiliary maljunction; SD, standard deviation; DSS, disease-specific survival. Statistical significance was defined as a P-value of <0.05.

a Indicates statistical significance.

**Table II:** Univariate analysis for prognostic factors of GBC.

Variables	No.	Median survival (month)	5-year survival rate	P-value
miR-155 expression				
High	13	25	0.35	0.01^a^
Low	13	45	0.85	
Age (years)				
≥65	13	27	0.47	0.44
<64	13	33	0.67	
Gender				
Male	11	51	0.68	0.63
Female	15	19	0.48	
PBM				
Present	9	19	0.68	0.35
Absent	17	27	0.51	
T category				
Tis-T2	23	27	0.57	0.99
T3-T4	3	17	0.50	
Nodal status				
N0	21	39	0.69	<0.0001^a^
N1	5	7	0.00	
UICC stage				
0-IIA	20	33	0.67	0.03^a^
IIB-IV	6	12	0.20	
Lymphatic invasion				
Positive	13	19	0.21	0.005^a^
Negative	13	81	0.90	
Vessel invasion				
Positive	12	20	0.40	0.07
Negative	14	33	0.69	
Perineural invasion				
Positive	6	15	0.30	0.26
Negative	20	33	0.63	

Statistical significance was defined as a P-value of <0.05.

a Indicates statistical significance.

**Table III:** Multivariate analysis for prognostic factors of GBC.

Variables	Hazard ratio	95% CI	P-value
High miR-155 expression	1.3	0.1–10	0.77
Nodal status (N1)	6.5	0.0–14	0.45
UICC stage (IIB-IV)	NA	NA	0.63
Lymphatic invasion	9.6	1.3–189	0.03^a^

miR, microRNA; GBC, gallbladder cancer; NA, not available; CI, confidence interval. Statistical significance was defined as a P-value of <0.05.

a Indicates statistical significance.

**Table IV:** Multivariate analysis for prognostic factors of GBCs at stages 0-IA.

Variables	Hazard ratio	95% CI	P-value
High miR-155 expression	9.9	1.10–294	0.03^a^
Lymphatic invasion	1.4	0.24–6.26	0.64
Vessel invasion	4.4	0.29–131	0.28

miR, microRNA; GBC, gallbladder cancer; CI, confidence interval. Statistical significance was defined as a P-value of <0.05.

a Indicates statistical significance.
